# Supplementary material for: Anti-Inflammatory Effects of Heliangin from Jerusalem Artichoke (Helianthus tuberosus) Leaves Might Prevent Atherosclerosis
Source: Biomolecules. 2022 Jan 6;12(1):91. doi: 10.3390/biom12010091 (PMC8774036; doi:10.3390/biom12010091)
Supplement: Supplementary file 1 [file biomolecules-12-00091-s001.zip › biomolecules-1523959-supplementary.pdf]

Supplementary Information

Anti-inflammatory effects of heliangin from Jerusalem artichoke (*Helianthus tuberosus*) leaves might prevent atherosclerosis

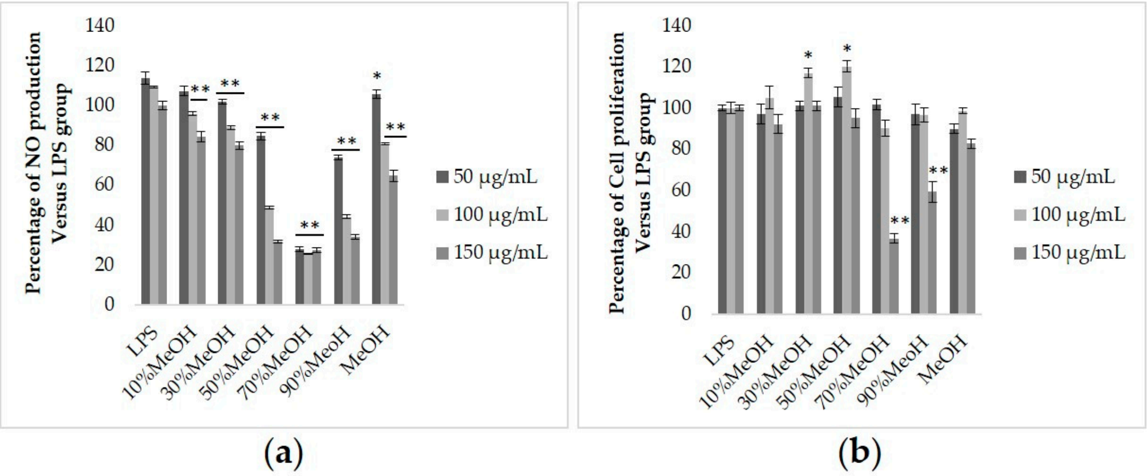

Figure S1. Effects of fractions separated using Sep-PaktC18 on RAW 264.7 cells stimulated with LPS. (**a**) Inhibition of NO production. (**b**) Cell proliferation. Values are shown as mean ± SD ($n = 4$) *$p < 0.05$ and **$p < 0.001$ *vs.* LPS (ANOVA with post hoc Dunnett tests).

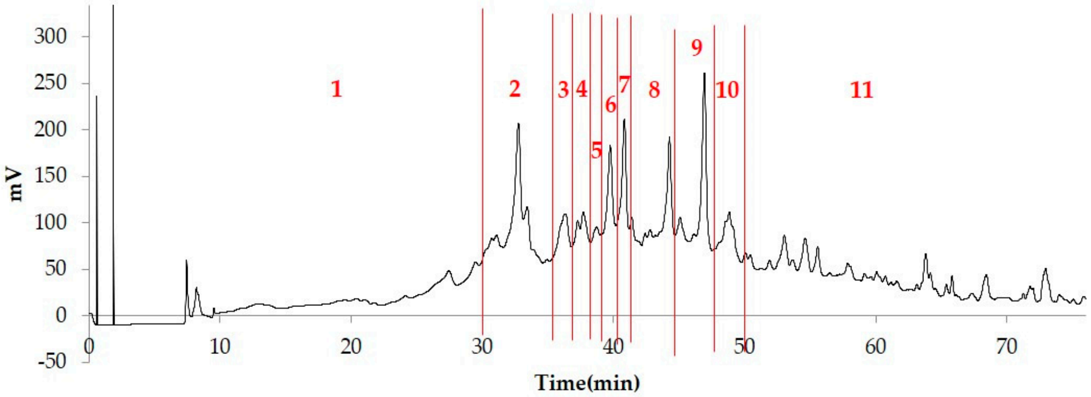

Figure S2. Compounds separated by HPLC from 50% methanol fraction.

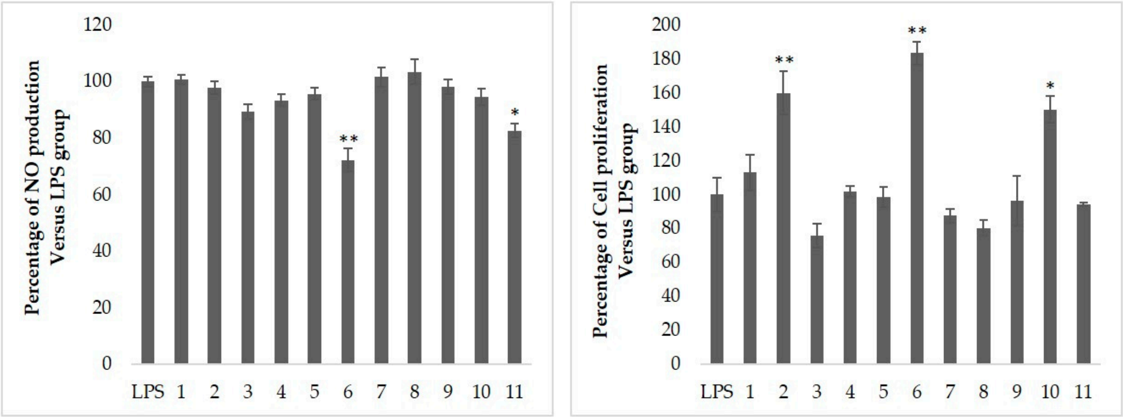

(**a**) (**b**)

Figure S3. Effects of fractions separated by HPLC in RAW 264.7 cells stimulated with LPS. (**a**) Inhibition of NO production. (**b**) Cell proliferation. Values are shown as mean ± SD (n = 4) *p < 0.01 and **p < 0.001 *vs*. LPS (ANOVA with post hoc Dunnett tests).

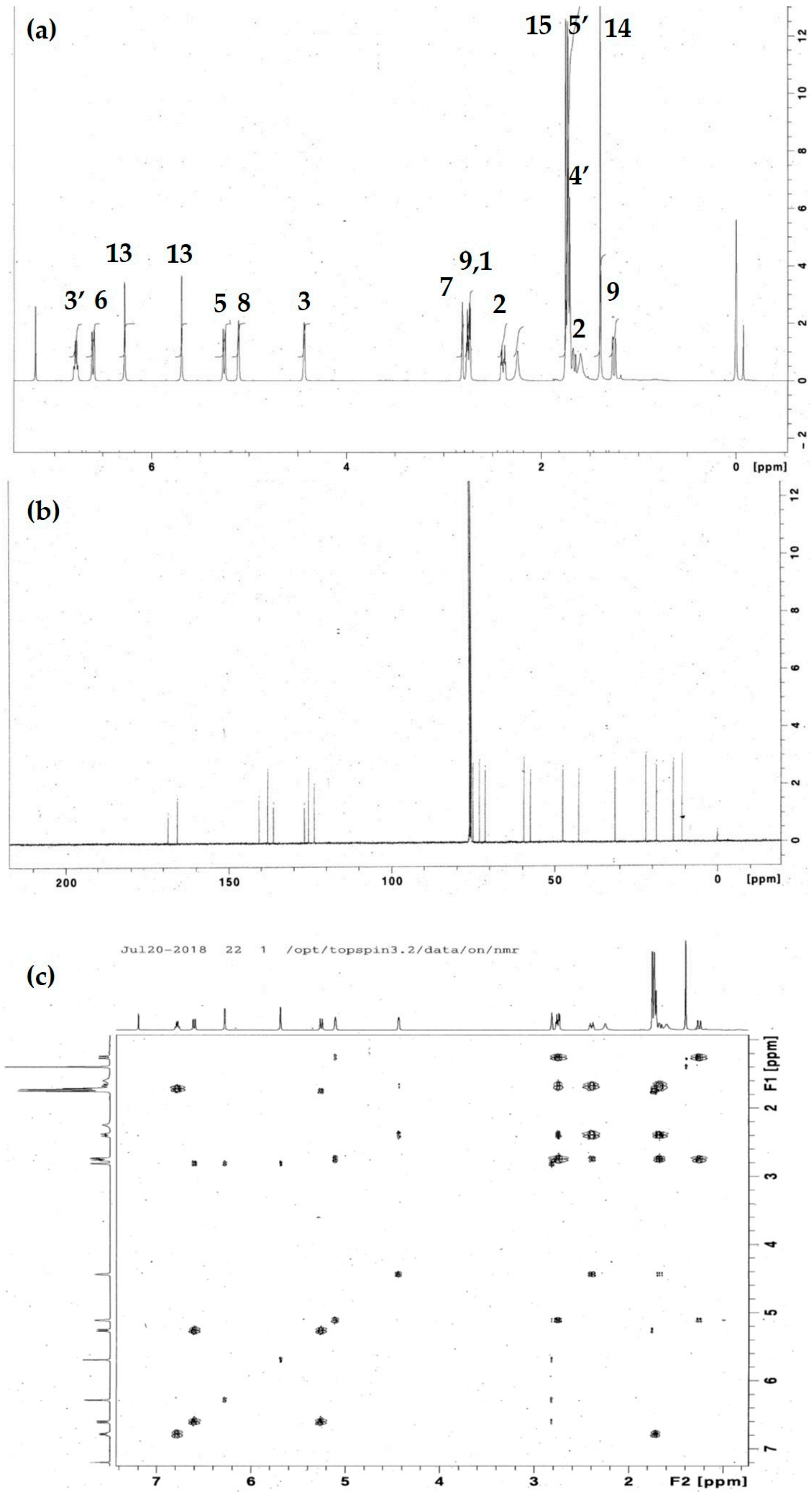

(a)
15
5′
14
4′
13
13
9,1
3′
6
5
8
3
7
2
9
2
[ppm]
(b)
[ppm]
(c)
Jul20-2018 22 1 /opt/topspin3.2/data/on/nmr
F1 [ppm]
F2 [ppm]

Figure S4. The one-dimensional (a) 1H and (b) 13C and (c) two-dimensional COSY spectra of heliangin.

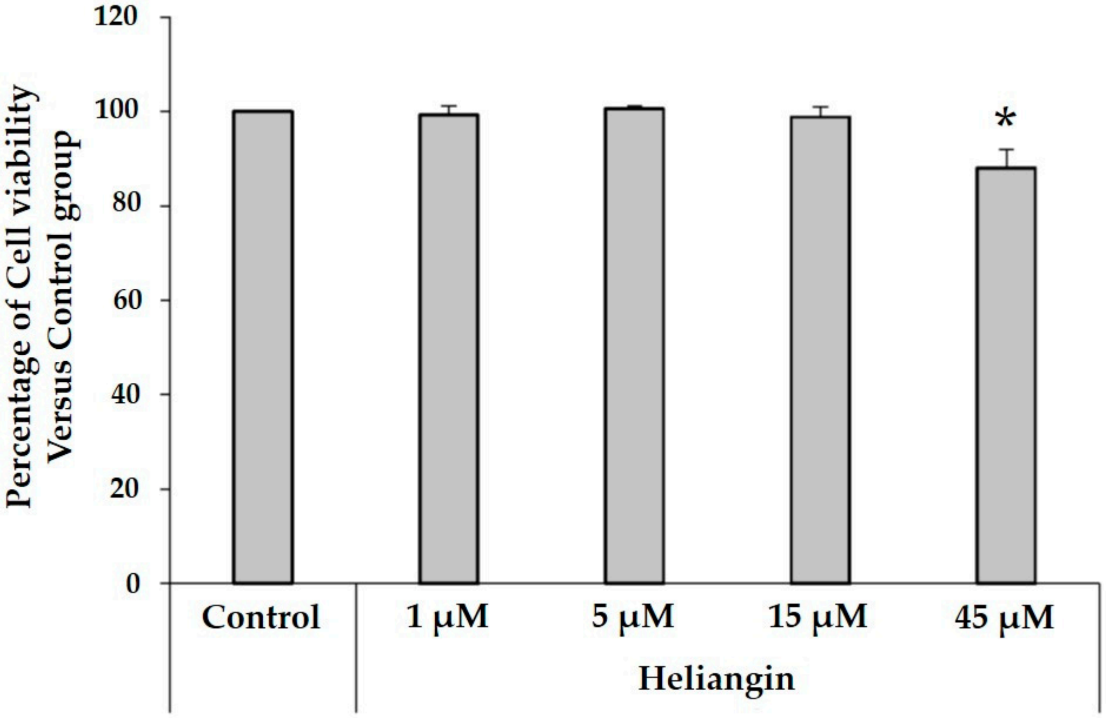

Figure S5. Effect of heliangin on VEC viability.

Vascular endothelial cells were treated with heliangin at 1, 5, 15 and 45 μM for 24 h. The cell viability was determined by trypan blue staining assay. Values are shown as means ± SD (n = 4) $P < 0.05$ vs. *Control (ANOVA with post hoc Dunnett tests).

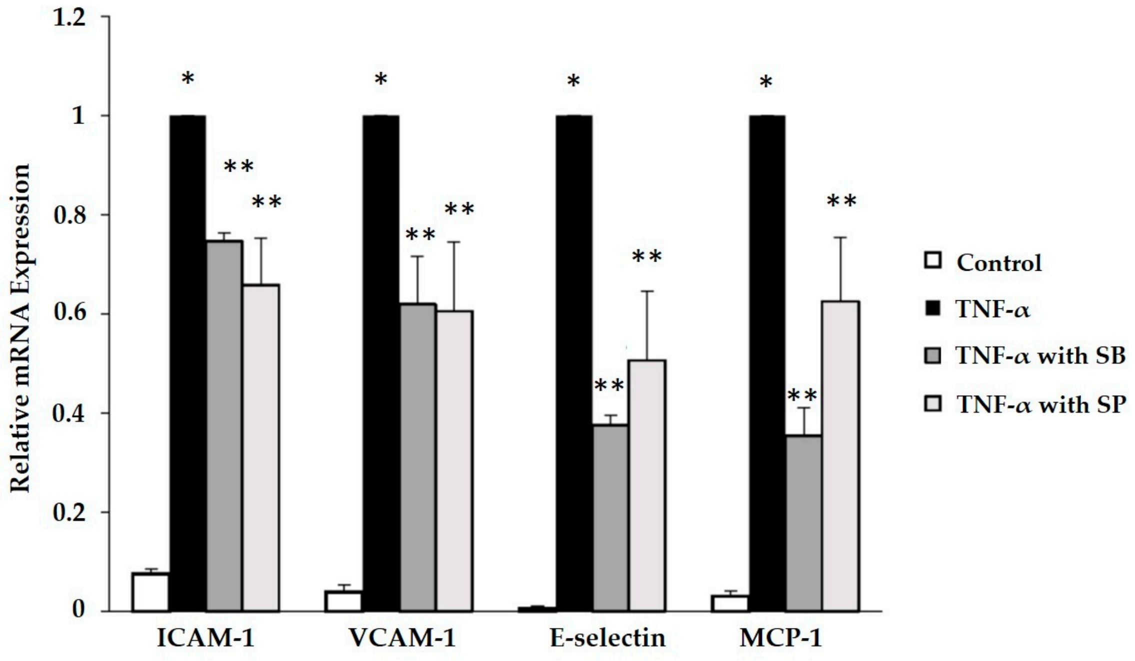

Figure S6. Inhibition of TNF-*α* in VECs.

Vascular endothelial cells were treated with 10 μM SB20358015 (SB) and 10 μM SP600125 (SP) for 1 h. Then, VECs were incubated with 2 ng/mL TNF-*α* for 3 h. The mRNA expression levels of ICAM, VCAM, E-selectin and MCP-1 were measured. Values are shown as means ± SD (n = 4) *$p < 0.05$ vs. Control and † $p < 0.05$ vs. TNF-*α* (ANOVA with Tukey`s Test).

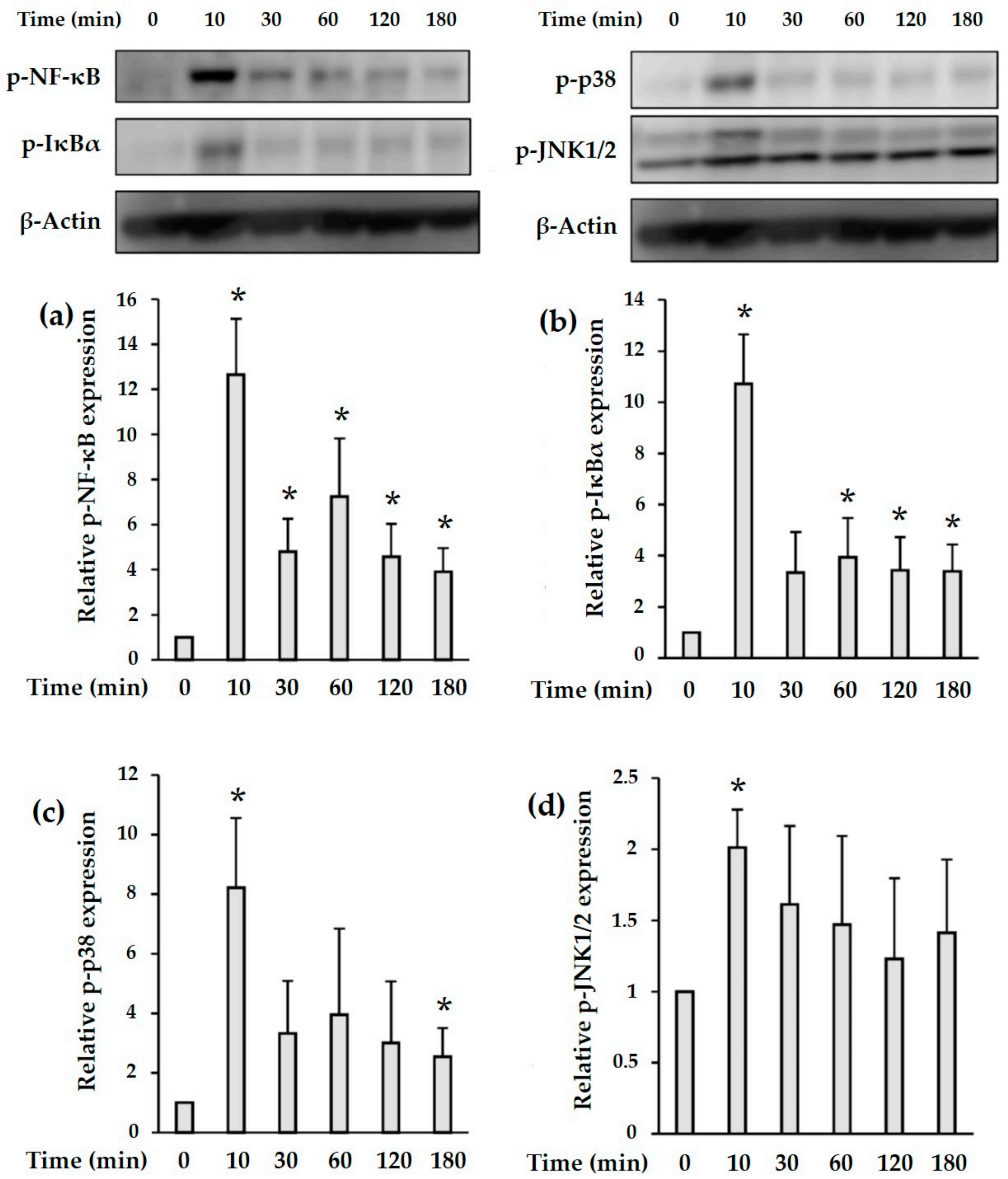

Figure S7. Effects of TNF-α on activation of inflammatory signaling pathways in VECs

Vascular endothelial cells were treated with 1 ng/mL TNF-α for 10, 30, 60, 120 and 180 min. The levels of (a) p-NF-κB, (b) p-IκBα, (c) p-p38 MAPK (p-p38) and (d) p-JNK1/2 were analyzed by western blotting. Values are shown as means ± SD (n = 5) *$p < 0.05$ vs. 0 min (ANOVA with post hoc Dunnett tests).
